# Supplementary material for: Meta-analysis of randomised controlled trials testing behavioural interventions to promote household action on climate change
Source: Nat Commun. 2019 Oct 4;10:4545. doi: 10.1038/s41467-019-12457-2 (PMC6778105; doi:10.1038/s41467-019-12457-2)
Supplement: Supplementary file 1 — Supplementary Information [file 41467_2019_12457_MOESM1_ESM.pdf]

Supplementary Information

Meta-analysis of randomised controlled trials testing behavioural interventions to promote household action on climate change

Nisa et al.

Supplementary Figure 1 -- 2

Supplementary Table 1 -- 3

Supplementary References -- 5

Supplementary Figures

Supplementary Figure 1. Prisma Flow

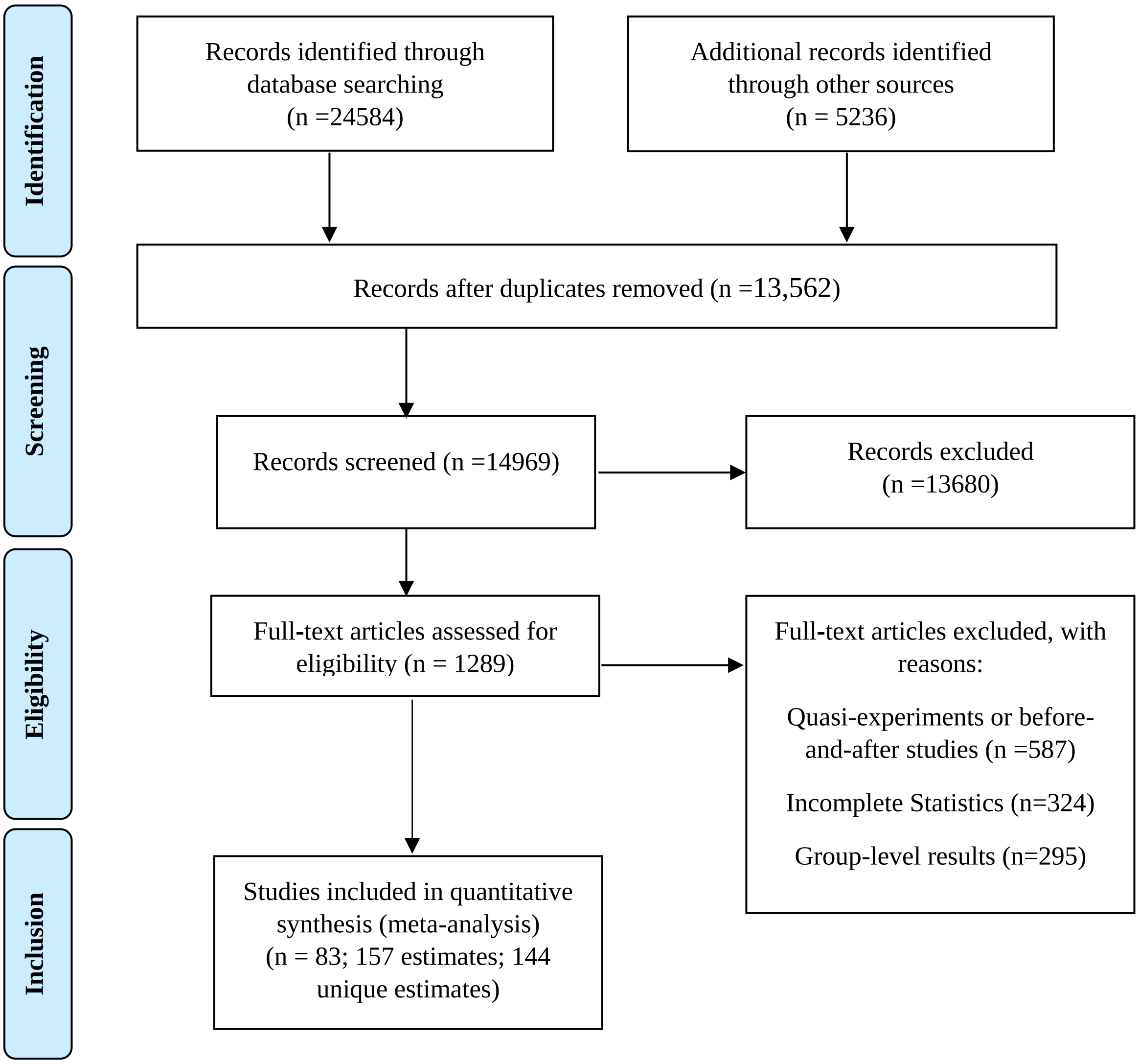

From: Moher D, Liberati A, Tetzlaff J, Altman DG, The PRISMA Group (2009). Preferred Reporting Items for Systematic Reviews and Meta-Analyses: The PRISMA Statement. PLoS Med 6(7): e1000097. doi:10.1371/journal.pmed1000097. For more information www.prisma-statement.org

Supplementary Tables

Supplementary Table 1. PRISMA STATEMENT

The 27 checklist items represent the best practices for reporting the content of systematic reviews and meta-analysis, which include the title, abstract, methods, results, discussion and funding (From: Moher D, Liberati A, Tetzlaff J, Altman DG, The PRISMA Group (2009). Preferred Reporting Items for Systematic Reviews and Meta-Analyses: The PRISMA Statement. PLoS Med 6(7): e1000097)

Section/topic	#	Checklist item	Reported on page #
**TITLE**			
Title	1	Identify the report as a systematic review, meta-analysis, or both.	1
**ABSTRACT**			
Structured summary	2	Provide a structured summary including, as applicable: background; objectives; data sources; study eligibility criteria, participants, and interventions; study appraisal and synthesis methods; results; limitations; conclusions and implications of key findings; systematic review registration number.	2
**INTRODUCTION**			
Rationale	3	Describe the rationale for the review in the context of what is already known.	3
Objectives	4	Provide an explicit statement of questions being addressed with reference to participants, interventions, comparisons, outcomes, and study design (PICOS).	3-4
**METHODS**			
Protocol and registration	5	Indicate if a review protocol exists, if and where it can be accessed (e.g., Web address), and, if available, provide registration information including registration number.	N.A.
Eligibility criteria	6	Specify study characteristics (e.g., PICOS, length of follow-up) and report characteristics (e.g., years considered, language, publication status) used as criteria for eligibility, giving rationale.	3-4, 12
Information sources	7	Describe all information sources (e.g., databases with dates of coverage, contact with study authors to identify additional studies) in the search and date last searched.	12
Search	8	Present full electronic search strategy for at least one database, including any limits used, such that it could be repeated.	12, SI p.17
Study selection	9	State the process for selecting studies (i.e., screening, eligibility, included in systematic review, and, if applicable, included in the meta-analysis).	12, SI p.17
Data collection process	10	Describe method of data extraction from reports (e.g., piloted forms, independently, in duplicate) and any processes for obtaining and confirming data from investigators.	12
Data items	11	List and define all variables for which data were sought (e.g., PICOS, funding sources) and any assumptions and simplifications made.	12, SI p.18-22
Risk of bias in individual studies	12	Describe methods used for assessing risk of bias of individual studies (including specification of whether this was done at the	4-6, 12-13

		study or outcome level), and how this information is to be used in any data synthesis.	
Summary measures	13	State the principal summary measures (e.g., risk ratio, difference in means).	2-3, 5, 12
Synthesis of results	14	Describe the methods of handling data and combining results of studies, if done, including measures of consistency (e.g., I^2) for each meta-analysis.	5, 12-13, SI 18-22
Risk of bias across studies	15	Specify any assessment of risk of bias that may affect the cumulative evidence (e.g., publication bias, selective reporting within studies).	5, 12-13, SI 18-22
Additional analyses	16	Describe methods of additional analyses (e.g., sensitivity or subgroup analyses, meta-regression), if done, indicating which were pre-specified.	5, 12-13, SI 18-22
**RESULTS**			
Study selection	17	Give numbers of studies screened, assessed for eligibility, and included in the review, with reasons for exclusions at each stage, ideally with a flow diagram.	4, 12, SI p.17
Study characteristics	18	For each study, present characteristics for which data were extracted (e.g., study size, PICOS, follow-up period) and provide the citations.	SI p.18-22
Risk of bias within studies	19	Present data on risk of bias of each study and, if available, any outcome level assessment (see item 12).	4-6, 12-13
Results of individual studies	20	For all outcomes considered (benefits or harms), present, for each study: (a) simple summary data for each intervention group (b) effect estimates and confidence intervals, ideally with a forest plot.	SI Database
Synthesis of results	21	Present results of each meta-analysis done, including confidence intervals and measures of consistency.	5
Risk of bias across studies	22	Present results of any assessment of risk of bias across studies (see Item 15).	5-7, 12-13
Additional analysis	23	Give results of additional analyses, if done (e.g., sensitivity or subgroup analyses, meta-regression [see Item 16]).	5-7
**DISCUSSION**			
Summary of evidence	24	Summarize the main findings including the strength of evidence for each main outcome; consider their relevance to key groups (e.g., healthcare providers, users, and policy makers).	1, 5-9
Limitations	25	Discuss limitations at study and outcome level (e.g., risk of bias), and at review-level (e.g., incomplete retrieval of identified research, reporting bias).	10-12
Conclusions	26	Provide a general interpretation of the results in the context of other evidence, and implications for future research.	9-12
**FUNDING**			
Funding	27	Describe sources of funding for the systematic review and other support (e.g., supply of data); role of funders for the systematic review.	14

Supplementary References

1. Aittasalo, M., Rinne, M., Pasanen, M., Kukkonen-Harjula, K., & Vasankari, T. (2012). Promoting walking among office employees―evaluation of a randomized controlled intervention with pedometers and e-mail messages. *BMC Public Health*, *12*(1), 403.
2. Allcott, H. (2011). Social norms and energy conservation. Journal of Public Economics, 95(Special Issue: The Role of Firms in Tax Systems), 1082-1095. doi:10.1016/j.jpubeco.2011.03.003
3. Allcott, H., & Sweeney, R. L. (2017). The role of sales agents in information disclosure: evidence from a field experiment. *Management Science*, *63*(1), 21-39.
4. Anderson, C. D., & Claxton, J. D. (1982). Barriers to consumer choice of energy efficient products. *Journal of Consumer Research*, *9*(2), 163-170.
5. Anderson, K., Song, K., Lee, S., Krupka, E., Lee, H., & Park, M. (2017). Longitudinal analysis of normative energy use feedback on dormitory occupants. *Applied energy*, *189*, 623-639.
6. Ascensio, O. I., & Delmas, M. A. (2015). Nonprice incentives and energy conservation. Proceedings of the National Academy of Sciences, 201401880.
7. Baca-Motes, K., Brown, A., Gneezy, A., Keenan, E. A., & Nelson, L. D. (2013). Commitment and behaviour change: Evidence from the field. Journal of Consumer Research, 39(5), 1070-1084.
8. Bamberg, S. (2006). Is a residential relocation a good opportunity to change people's travel behaviour? Results from a theory-driven intervention study. *Environment and behaviour*, *38*(6), 820-840.
9. Bamberg, S., & Rees, J. (2017). The impact of voluntary travel behaviour change measures–A meta-analytical comparison of quasi-experimental and experimental evidence. *Transportation Research Part A: Policy and Practice*, *100*, 16-26.
10. Bapuji, H., Hora, M., & Saeed, A. M. (2012). Intentions, intermediaries, and interaction: Examining the emergence of routines. *Journal of Management Studies*, *49*(8), 1586-1607.
11. Becker, L. J. (1978). Joint effect of feedback and goal setting on performance: A field study of residential energy conservation. Journal of applied psychology, 63(4), 428.
12. Bohner, G., & Schlüter, L. E. (2014). A room with a viewpoint revisited: Descriptive norms and hotel guests' towel reuse behaviour. PloS one, 9(8), e104086.
13. Bryce, W. J., Day, R., & Olney, T. J. (1997). Commitment approach to motivating community recycling: New Zealand curbside trial. The Journal of Consumer Affairs, 27-52.
14. Burn, S. M. (1991). Social Psychology and the Stimulation of Recycling Behaviours: The Block Leader Approach. Journal of Applied Social Psychology, 21(8), 611.
15. Burn, S. M., & Oskamp, S. (1986). Increasing community recycling with persuasive communication and public commitment. *Journal of Applied Social Psychology*, *16*(1), 29-41.
16. Carrico, A. R., & Riemer, M. (2011). Motivating energy conservation in the workplace: An evaluation of the use of group-level feedback and peer education. Journal of Environmental Psychology, 311-13. doi:10.1016/j.jenvp.2010.11.004
17. Carroll, J., Denny, E., & Lyons, S. (2016). The effects of energy cost labelling on appliance purchasing decisions: trial results from Ireland. *Journal of consumer policy*, *39*(1), 23-40.
18. Chen, V. L., Delmas, M. A., Locke, S. L., & Singh, A. (2017). Information strategies for energy conservation: A field experiment in India. *Energy Economics*, *68*, 215-227.
19. Christina, S., Dainty, A., Daniels, K., Tregaskis, O., & Waterson, P. (2017). Shut the fridge door! HRM alignment, job redesign and energy performance. *Human Resource Management Journal*, *27*(3), 382-402.
20. Clayton, M., & Nesnidol, S. (2017). Reducing electricity use on campus: The use of prompts, feedback, and goal setting to decrease excessive classroom lighting. *Journal of Organizational Behaviour Management*, *37*(2), 196-206.
21. Cobern, M. K., Porter, B. E., Leeming, F. C., & Dwyer, W. O. (1995). The effect of commitment on adoption and diffusion of grass cycling. Environment and Behaviour, 27(2), 213-232.
22. Costa, D. L., & Kahn, M. E. (2013). Energy conservation "nudges" and environmentalist ideology: evidence from a randomized residential electricity field experiment. Journal of the European Economic Association, 11(3), 680-702.
23. Datta, S., Miranda, J. J., Zoratto, L., Calvo-González, O., Darling, M., & Lorenzana, K. (2015). *A behavioural approach to water conservation: evidence from Costa Rica*. The World Bank

https://openknowledge.worldbank.org/bitstream/handle/10986/22156/A0behavioural0a0ence0from0Costa0Rica.pdf?sequence=1

24. Delmas, M. A., & Lessem, N. (2014). Saving power to conserve your reputation? The effectiveness of private versus public information. Journal of Environmental Economics and Management, 67353-370. doi:10.1016/j.jeem.2013.12.009
25. Dickerson, C. A. (1992). Using Cognitive Dissonance to Encourage Water Conservation. Journal of Applied Social Psychology, 22(11), 841-854.
26. Dolan, P., & Metcalfe, R. (2013). Neighbors, knowledge, and nuggets: two natural field experiments on the role of incentives on energy conservation.
27. Eriksson, L., Garvill, J., & Nordlund, A. M. (2008). Interrupting habitual car use: The importance of car habit strength and moral motivation for personal car use reduction. *Transportation Research Part F: Traffic Psychology and Behaviour*, *11*(1), 10-23.
28. Ferraro, P. J., & Price, M. K. (2013). Using nonpecuniary strategies to influence behaviour: evidence from a large-scale field experiment. Review of Economics and Statistics, 95(1), 64-73.
29. Fielding, K. S., Spinks, A., Russell, S., McCrea, R., Stewart, R., & Gardner, J. (2013). An experimental test of voluntary strategies to promote urban water demand management. Journal of Environmental Management, 114343-351. doi:10.1016/j.jenvman.2012.10.027
30. Friis, R., Skov, L. R., Olsen, A., Appleton, K. M., Saulais, L., Dinnella, C. & Perez-Cueto, F. J. (2017). Comparison of three nudge interventions (priming, default option, and perceived variety) to promote vegetable consumption in a self-service buffet setting. *PloS one*, *12*(5), e0176028.
31. Fujii, S., & Taniguchi, A. (2005). Reducing family car-use by providing travel advice or requesting behavioural plans: An experimental analysis of travel feedback programs. *Transportation Research Part D: Transport and Environment*, *10*(5), 385-393 [duplicate Taniguchi, A., & Fujii, S. (2007). Promoting public transport using marketing techniques in mobility management and verifying their quantitative effects. *Transportation*, *34*(1), 37.]
32. Garvill, J., Marell, A., & Nordlund, A. (2003). Effects of increased awareness on choice of travel mode. *Transportation*, *30*(1), 63-79.
33. Goldstein, N. J., Cialdini, R. B., & Griskevicius, V. (2008). A room with a viewpoint: Using social norms to motivate environmental conservation in hotels. Journal of consumer Research, 35(3), 472-482.
34. Goldstein, N. J., Griskevicius, V., & Cialdini, R. B. (2011). Reciprocity by proxy: A novel influence strategy for stimulating cooperation. *Administrative Science Quarterly*, *56*(3), 441-473.
35. Goodhew, J., Pahl, S., Auburn, T., & Goodhew, S. (2015). Making heat visible: promoting energy conservation behaviours through thermal imaging. *Environment and Behaviour*, *47*(10), 1059-1088.
36. Goodman, A., Panter, J., Sharp, S. J., & Ogilvie, D. (2013). Effectiveness and equity impacts of town-wide cycling initiatives in England: a longitudinal, controlled natural experimental study. *Social Science & Medicine*, *97*, 228-237.
37. Handgraaf, M. J., de Jeude, M. A. V. L., & Appelt, K. C. (2013). Public praise vs. private pay: Effects of rewards on energy conservation in the workplace. Ecological Economics, 86, 86-92.
38. Hemmingsson, E., Udden, J., Neovius, M., Ekelund, U., & Rössner, S. (2009). Increased physical activity in abdominally obese women through support for changed commuting habits: a randomized clinical trial. *International journal of obesity*, *33*(6), 645.
39. Holland, R. W., Aarts, H., & Langendam, D. (2006). Breaking and creating habits on the working floor: A field-experiment on the power of implementation intentions. Journal of Experimental Social Psychology, 42776-783. doi:10.1016/j.jesp.2005.11.006
40. Houde, S., Todd, A., Sudarshan, A., Flora, J. A., & Armel, K. C. (2013). Real-time feedback and electricity consumption: A field experiment assessing the potential for savings and persistence. The Energy Journal, 34(1), 87.
41. Hsieh, H. S., Kanda, Y., & Fujii, S. (2017). Reducing car use by volitional strategy of action and coping planning enhancement. *Transportation research part F: traffic psychology and behaviour*, *47*, 163-175.
42. Hutton, R. B., & Ahtola, O. T. (1991). Consumer response to a five-year campaign to combat air pollution. *Journal of Public Policy & Marketing*, 242-256.

43. Jeong, S. H., Gulbinas, R., Jain, R. K., & Taylor, J. E. (2014). The impact of combined water and energy consumption eco-feedback on conservation. Energy and Buildings, 80, 114-119.
44. Kallbekken, S., & Sælen, H. (2013). 'Nudging'hotel guests to reduce food waste as a win–win environmental measure. *Economics Letters*, *119*(3), 325-327.
45. Kallbekken, S., Sælen, H., & Hermansen, E. A. (2013). Bridging the energy efficiency gap: A field experiment on lifetime energy costs and household appliances. *Journal of Consumer Policy, 36*(1), 1-16.
46. Katzev, R. D., & Pardini, A. U. (1987). The comparative effectiveness of reward and commitment approaches in motivating community recycling. Journal of Environmental Systems, 17(2), 93-113.
47. Katzev, R., Cooper, L., & Fisher, P. (1980). The effect of feedback and social reinforcement on residential electricity consumption. Journal of Environmental Systems, 10(3), 215-227.
48. Kendel, A., Lazaric, N., & Maréchal, K. (2017). What do people 'learn by looking'at direct feedback on their energy consumption? Results of a field study in Southern France. *Energy Policy, 108,* 593-605.
49. Kongsbak, I., Skov, L. R., Nielsen, B. K., Ahlmann, F. K., Schaldemose, H., Atkinson, L., & Pérez-Cueto, F. J. (2016). Increasing fruit and vegetable intake among male university students in an ad libitum buffet setting: A choice architectural nudge intervention. *Food quality and preference*, *49*, 183-188.
50. Kurz, T., Donaghue, N., & Walker, I. (2005). Utilizing a Social-Ecological Framework to Promote Water and Energy Conservation: A Field Experiment. Journal of Applied Social Psychology, 35(6), 1281-1300.
51. Lynham, J., Nitta, K., Saijo, T., & Tarui, N. (2016). Why does real-time information reduce energy consumption? *Energy Economics*, *54*, 173-181.
52. Mair, J., & Bergin-Seers, S. (2010). The effect of interventions on the environmental behaviour of Australian motel guests. *Tourism and Hospitality Research*, *10*(4), 255-268.
53. Matsukawa, I. (2004). The Effects of Information on Residential Demand for Electricity. The Energy Journal, 25(1), 1-18.
54. McCaul, K. D., & Kopp, J. T. (1982). Effects of Goal Setting and Commitment on Increasing Metal Recycling. Journal of Applied Psychology, 67(3), 377-379.
55. Mutrie, N., Carney, C., Blamey, A., Crawford, F., Aitchison, T., & Whitelaw, A. (2002). "Walk in to Work Out": a randomised controlled trial of a self-help intervention to promote active commuting. *Journal of Epidemiology & Community Health*, *56*(6), 407-412.
56. Nolan, J. M., Schultz, P. W., Cialdini, R. B., Goldstein, N. J., & Griskevicius, V. (2008). Normative social influence is underdetected. Personality and social psychology bulletin, 34(7), 913-923.
57. Pardini, A. U., & Katzev, R. D. (1983). The effect of strength of commitment on newspaper recycling. *Journal of Environmental Systems*, *13*(3), 245-254.
58. Qi, D., & Roe, B. E. (2017). Foodservice composting crowds out consumer food waste reduction behaviour in a dining experiment. *American Journal of Agricultural Economics*, *99*(5), 1159-1171.
59. Reese, G., Loew, K., & Steffgen, G. (2014). A towel less: Social norms enhance pro-environmental behaviour in hotels. The Journal of social psychology, 154(2), 97-100.
60. Rowland, D., DiGuiseppi, C., Gross, M., Afolabi, E., & Roberts, I. (2003). Randomised controlled trial of site specific advice on school travel patterns. *Archives of disease in childhood*, *88*(1), 8-11.
61. Sargeant, J., Carter, T., McSweeney, S., & Hughes, W. (2004a). Cambridgeshire Travel Choice Project: New Recruit. *Department for Transport, Cambridgeshire County Council, Cambridge University Hospitals NHS Foundation Trust, Cambridgeshire Travel for Work Partnership, Cambridge, UK, Final Report*.
62. Sargeant, J., Carter, T., McSweeney, S., & Hughes, W. (2004b). Cambridgeshire Travel Choice Project: Addenbrooke's Hospital. *Department for Transport, Cambridgeshire County Council, Cambridge University Hospitals NHS Foundation Trust, Cambridgeshire Travel for Work Partnership, Cambridge, UK, Final Report*.
63. Sargeant, J., Carter, T., McSweeney, S., & Hughes, W. (2004c). Cambridgeshire Travel Choice Project: Car Park. *Department for Transport, Cambridgeshire County Council, Cambridge University Hospitals NHS Foundation Trust, Cambridgeshire Travel for Work Partnership, Cambridge, UK, Final Report*.

64. Schleich, J., Klobasa, M., Gölz, S., & Brunner, M. (2013). Effects of feedback on residential electricity demand—Findings from a field trial in Austria. Energy Policy, 61, 1097-1106.
65. Schultz, P. W. (1998). Changing Behaviour with Normative Feedback Interventions: A Field Experiment on Curbside Recycling. Basic & Applied Social Psychology, 21(1), 25-36.
66. Schultz, P. W., Nolan, J. M., Cialdini, R. B., Goldstein, N. J., & Griskevicius, V. (2008). The Constructive, Destructive, and Reconstructive Power of Social Norms. Psychological Science (Wiley-Blackwell), 18(5), 429-434. doi:10.1111/j.1467-9280.2007.01917.x
67. Schultz, P. W., Estrada, M., Schmitt, J., Sokoloski, R., & Silva-Send, N. (2015). Using in-home displays to provide smart meter feedback about household electricity consumption: A randomized control trial comparing kilowatts, cost, and social norms. Energy, 90, 351-358.
68. Schultz, P. W., Messina, A., Tronu, G., Limas, E. F., Gupta, R., & Estrada, M. (2016). Personalized normative feedback and the moderating role of personal norms a field experiment to reduce residential water consumption. Environment and Behaviour, 0013916514553835.
69. Schwartz, D., Fischhoff, B., Krishnamurti, T., & Sowell, F. (2013). The Hawthorne effect and energy awareness. Proceedings of the National Academy of Sciences, 110(38), 15242-15246.
70. Seligman, C., & Darley, J. M. (1976). Feedback as a means of decreasing residential energy consumption. Journal of Applied Psychology, 62(4), 363.
71. Seyranian, V., Sinatra, G. M., & Polikoff, M. S. (2015). Comparing communication strategies for reducing residential water consumption. Journal of Environmental Psychology, 4181-90. doi:10.1016/j.jenvp.2014.11.009
72. Spaccarelli, S., Zolik, E., & Jason, L. A. (1989). Effects of verbal prompting and block characteristics on participation in curbside newspaper recycling. *Journal of Environmental Systems*, *19*(1), 45-57.
73. Sparkman, G., & Walton, G. M. (2017). Dynamic norms promote sustainable behaviour, even if it is counternormative. *Psychological science*, *28*(11), 1663-1674.
74. Sussman, R., & Gifford, R. (2011). Be the Change You Want to See Modeling Food Composting in Public Places. Environment and Behaviour, 45(3), 323-343.
75. Terrier, L., & Marfaing, B. (2015). Using social norms and commitment to promote proenvironmental behaviour among hotel guests. Journal of Environmental Psychology, 44, 10-15.
76. Tertoolen, G., Van Kreveld, D., & Verstraten, B. (1998). Psychological resistance against attempts to reduce private car use. *Transportation Research Part A: Policy and Practice*, *32*(3), 171-181. Thompson, S. C., & Stoutemyer, K. (1991). Water Use as a Commons Dilemma The Effects of Education that Focuses on Long-Term Consequences and Individual Action. Environment and Behaviour, 23(3), 314-333.
77. Thondhlana, G., & Kua, H. W. (2016). Promoting household energy conservation in low-income households through tailored interventions in Grahamstown, South Africa. *Journal of Cleaner Production*, *131*, 327-340.
78. Tørnblad, S. H., Kallbekken, S., Korneliussen, K., & Mideksa, T. K. (2014). Using mobility management to reduce private car use: Results from a natural field experiment in Norway. *Transport policy*, *32*, 9-15.
79. Wansink, B., & Van Ittersum, K. (2013). Portion size me: Plate-size induced consumption norms and win-win solutions for reducing food intake and waste. *Journal of Experimental Psychology: Applied*, *19*(4), 320.
80. Wen, L. M., Fry, D., Merom, D., Rissel, C., Dirkis, H., & Balafas, A. (2008). Increasing active travel to school: are we on the right track? A cluster randomised controlled trial from Sydney, Australia. *Preventive medicine*, *47*(6), 612-618.
81. Werner, C. M., Turner, J., Shipman, K., Shawn Twitchell, F., Dickson, B. R., Bruschke, G. V., & von Bismarck, W. B. (1995). Commitment, behaviour, and attitude change: An analysis of voluntary recycling. Journal 0f Environmental Psychology, 15(Green Psychology), 197-208.
82. Winett, R. A., Neale, M. S., & Grier, H. C. (1979). Effects of self-monitoring and feedback on residential electricity consumption. Journal of Applied Behaviour Analysis, 12(2), 173-184.
