# Supplementary material for: Meta-analysis of randomised controlled trials testing behavioural interventions to promote household action on climate change
Source: Nat Commun. 2019 Oct 4;10:4545. doi: 10.1038/s41467-019-12457-2 (PMC6778105; doi:10.1038/s41467-019-12457-2)
Supplement: Supplementary file 3 — Description of Additional Supplementary Files [file 41467_2019_12457_MOESM3_ESM.pdf]

Description of Additional Supplementary Files

File Name: Supplementary Data 1

Description: Description of Included Papers
